# Reactive airways dysfunction syndrome following inhalation of hydrogen chloride vapor

**DOI:** 10.4322/acr.2021.266

**Published:** 2021-04-23

**Authors:** Vanessa Simioni Faria, Soraya Abou El Hosn Cordero da Silva, Julio Flávio Meirelles Marchini

**Affiliations:** 1 Universidade de São Paulo (USP), Hospital das Clínicas, Disciplina de Emergências Clínicas do Departamento de Clínica Médica, São Paulo, SP, Brasil.; 2 Universidade Federal de São Paulo (UNIFESP), Hospital São Paulo, Departamento de Pneumologia, São Paulo, SP, Brasil.

**Keywords:** Bronchial Hyperreactivity, Respiratory Insufficiency, Respiratory Distress Syndrome, Adult

## Abstract

Hydrogen chloride is available commercially as an anhydrous gas or an aqueous solution, hydrochloric acid. Exposure to this gas has been associated with the development of reactive airways dysfunction syndrome. However, there are few published reports. A 37-year-old woman developed progressive bronchospasm and acute respiratory failure after cleaning an enclosed space with an unknown concentration of hydrochloric acid gas from a cleaning substance. She had no prior history of asthma or atopy. Severe bronchospasm developed, leading to hypoxemia and diffuse interstitial infiltrates, necessitating orotracheal intubation and admission to the intensive care unit. Asthma-like symptoms such as cough, wheezing, and dyspnea; requiring bronchodilators, and repeated hospitalizations are persistent a year after the accident. Pulmonary function testing showed mild airflow obstruction.

## INTRODUCTION

Reactive airways dysfunction syndrome (RADS) is defined as acute and persistent respiratory symptoms and nonspecific bronchial hyperreactivity. It was first described by Brooks et al.^1^ to designate an asthma-like condition that may develop following exposure to toxic gases. This syndrome appears shortly after exposure to the causative agent. It is characterized by increased non-allergic airway hyperreactivity and asthma-like symptoms in a subject with no history of asthma prior to the exposure. This syndrome can occur after exposure to a variety of toxic gases, including hydrochloric acid (HCl), ammonia, and hydrogen sulfide. Patients may persist symptomatic even after a single exposure to the toxic gas.^2^ This syndrome appears to have an equal male to female incidence.^3^

HCl is a non-flammable and colorless liquid with a strong, pungent odor commercialized as a chemical product component used to clean and disinfect swimming pools.^4^ When present at a concentration of 25% or more, it fumes in the air and becomes hydrogen chloride gas.^5^ ^-^ ^7^ Its high-water solubility allows for rapid dissolution on mucous membranes after inhalation, normally causing low systemic toxicity. High concentrations of HCl appear to saturate the buffering capacities of the nasal mucosa.^6^ ^,^ ^8^ Once HCl is absorbed into the mucous layers and membranes, it dissociates into hydrogen and chloride ions. The hydrogen ions in contact with water produce hydronium ions, which rapidly react with organic molecules. That reaction is presumably responsible for the cellular injury.^8^ ^,^ ^9^ Meanwhile, the chloride ions are not sufficient to disturb the body’s electrolyte balance.^8^

Respiratory tract effects range from mild to moderate irritation at low concentrations (below 100ppm). At higher concentrations, greater than 500ppm, hydrogen chloride may cause tachypnea, shortness of breathing, chest tightness, wheeze/stridor, dyspnea, and hypoxemia.^9^ ^-^ ^11^ Some severely exposed patients may develop acute respiratory distress syndrome from direct damage to the respiratory cells at the alveolar level or indirectly through inflammation mediators.^10^

This is a case report of a housekeeper exposed to high HCl fumes concentrations that evolved with acute respiratory distress syndrome suggestive of RADS, a still not fully-known disorder as to prognosis and its long-term effects.

## CASE REPORT

A 37-year-old Caucasian woman arrived at the emergency facility with severe respiratory distress, associated with pleuritic pain and tachycardia. She denied any previous comorbidities or use of medications, except for a nine pack-year history of smoking. The patient reported she had applied a cleaning substance to wash a stone floor in a small and closed room. After a few minutes, she came back into this room to scrub the floor, and within a short period, she started to feel discomfort and dyspnea and left the room. Her dyspnea became progressively worse in the following two days when she decided to look for help.

**Figure 1:**
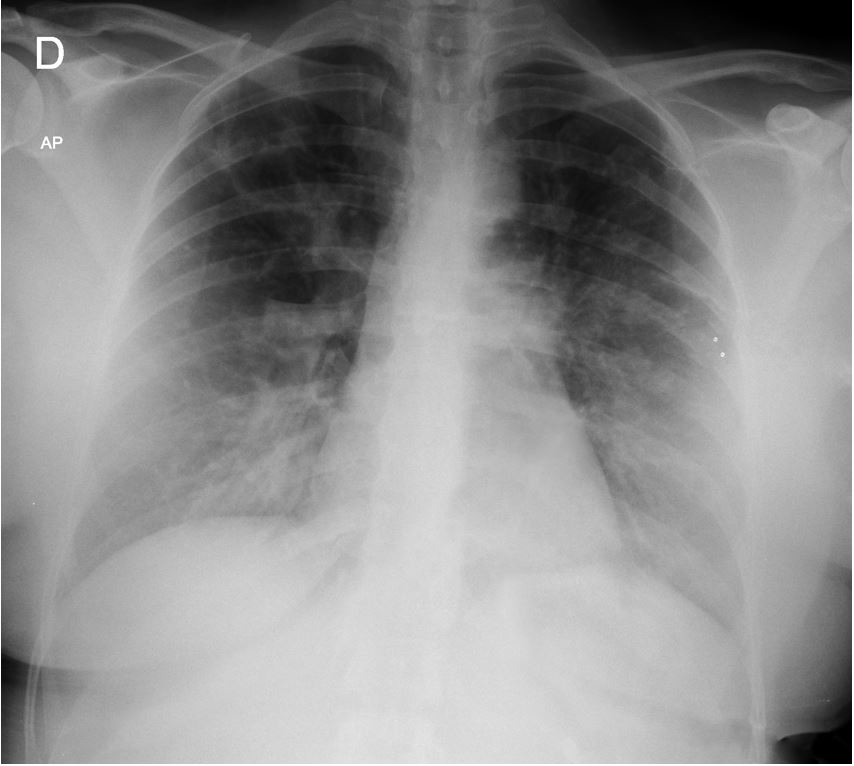
An anteroposterior chest radiograph at admission demonstrating bilateral interstitial infiltrates.

**Figure 2:**
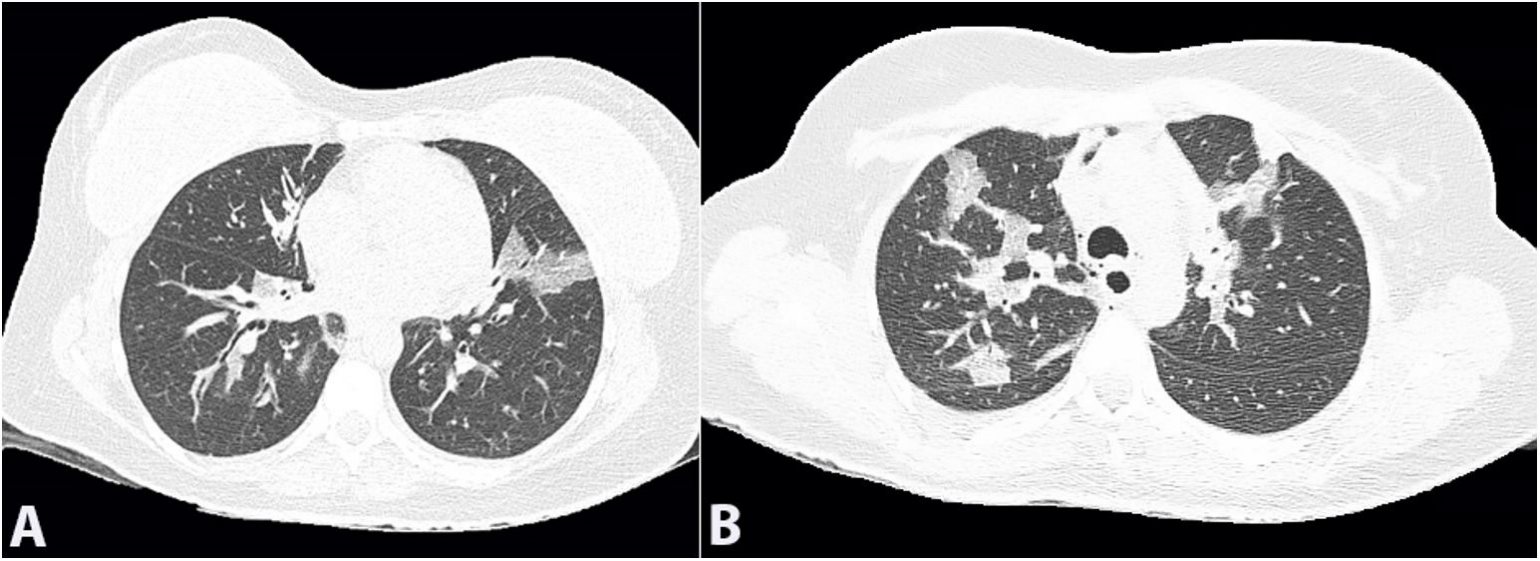
Chest CT images (lung window) in medium (A) and apical (B) axial planes show bilateral ground-glass opacities with consolidation areas involving all pulmonary lobes.

On admission, the patient had a respiratory rate of thirty-eight breaths per minute and diffuse wheezing and rhonchi. She was treated with three fenoterol cycles (2.5mg) and ipratropium (0.5mg) inhalation. Meanwhile, we identified she had used a cleaning substance composed mainly of hydrochloric acid (HCl). Her exams showed leukocytosis of 23.06x10^6^/L (RR: 4.0 - 11.0 x10^6^/L), composed of 88% neutrophils and 1.3% eosinophils; a C-reactive protein of 256mg/L (RR: below 5 mg/L), and the arterial blood gas collected with 35% of the fraction of inspired oxygen (FiO_2_) had a pH of 7.34 (RR: 7.35 - 7.45), the partial pressure of Oxygen (PaO_2_) of 74.5mmHg (RR: 80 - 100 mmHg), the partial pressure of carbon dioxide of 35.8mmHg (RR: 35 - 45 mmHg), bicarbonate of 19.2mmol/L (RR: 21 - 28 mmol/L), base excess of -5.2mmol/L (RR: -3 - +3 mmol/L), oxygen saturation of 93.6% and a PaO_2_/FiO_2_ ratio of 212. The chest radiograph showed bilateral interstitial infiltrates (Figure 1). The patient met the Berlin criteria^12^ for acute respiratory distress syndrome. Despite treatment with bronchodilators and non-invasive ventilation, the patient became progressively worse, and her respiratory rate increased to 55 breaths per minute (bpm). Orotracheal intubation proceeded after ketamine, propofol, and succinylcholine, and she was set up on mechanical ventilation using parameters for lung protection with a low tidal volume. She was admitted to the intensive care unit and rapidly improved within the following twenty-four hours. A chest computed tomography (CT) scan showed diffuse asymmetric bilateral ground-glass opacities (Figure 2). She was successfully extubated the next day. Non-invasive ventilation was necessary for the first 4 hours after extubation due to bronchospasm. Methylprednisolone 60mg intravenously was given for the first two days, followed by prednisone 40mg orally for four days. The patient presented a dry cough and slight limitation of physical activity. She denied any other symptoms and was discharged.

We evaluated her one year after her index admission. In the first three months, the patient continued to present shortness of breath, cough and wheezing, which could be triggered by minimal concentrations of irritants. Since her index admission, the patient had had four more admissions to the Emergency Department with respiratory complaints. Her clinical exam revealed no abnormality, and she stated she stopped smoking since the event. The spirometry data showed a forced expiratory volume in one second (FEV_1_)/ forced vital capacity (FVC) ratio of 0.79 (the predicted ratio for her was 0.83); reduced FEV_1_ (77% of predicted) and FVC (81% of predicted). There was pulmonary flow gain after bronchodilator use (Delta FEV1= 260ml and 12%), demonstrating a positive response to bronchodilator and a mild obstructive ventilatory disorder. In the body plethysmography, the total lung capacity (TLC) was 102% of predicted; and the thoracic gas volume (TGV) was 114% of predicted. The residual volume (RV) was at the upper limit of normality (137% of predicted), and there was a high RV/TLC value of 42 (135% of predicted), suggestive of air trapping. A normal diffusion capacity was seen (78% of predicted).

## DISCUSSION

In the late 1800s and first half of the 1900s, human studies of hydrogen chloride (HCl) exposure-response were performed in laboratories. Despite the remaining important data source, these reports are limited because the methodology used is less detailed than the current practice standards.^8^ There are no recent experimental human studies on HCl irritant effects except at very low concentrations (around 1.8ppm), and the last available report was published in 1992. Ten asthmatic individuals (female/male) were exposed to 0.8 or 1.8 ppm HCl for forty-five minutes. Pulmonary function tests performed immediately after the exposure were compared to baseline levels. No exposure-related effects were observed in subjective symptoms or pulmonary function tests.^13^

Inhaled HCl may cause functional and morphologic respiratory tract injuries, depending on the exposure concentration and duration. The maximum bearable concentration in prolonged exposure of humans was reported as 10ppm; however, 10-50ppm for an exposure of a few hours is tolerable.^8^ Respiratory tract effects in laboratory animals range from mild to moderate irritation at low concentrations (less than 100ppm) to nasal lesions at moderate concentrations (100-500 ppm) and pulmonary damage at high concentrations (greater than 500ppm), even with fairly short exposure periods. The concentration of 1000ppm and above cause unconsciousness, followed by death in thirty minutes to one hour.^5^ ^,^ ^8^

Because of its high-water solubility, most of the inhaled HCl gas is absorbed and neutralized by ammonia gas in the upper respiratory tract, explaining the occurrence of delayed respiratory irritation signs.^6^ ^,^ ^8^ ^,^ ^9^ ^,^ ^13^ In fact, little is known about the acid-base buffering capacity of respiratory mucous membranes and tissues.^8^

In a short-term study, three male baboons/groups were exposed for fifteen minutes to different HCl concentrations (0, 500, 5000, or 10000ppm).^14^ The results indicated that the increased respiratory rate following the exposure was dose-related. Higher doses were associated with an acutely decreased partial pressure of oxygen (PaO_2_). Hypoxemia can be caused by ventilation-perfusion mismatches. Gas exchange can be impaired by interstitial and alveolar edema. Swelling of type I respiratory cells and endothelial cells, which corresponds to additional thickening of the alveolar-capillary membrane, further impairs gas exchange. Arterial hypoxemia can also be worsened by alveoli collapse because of reduced surfactant production by the type II respiratory cells and surfactant proteins’ denaturation.^10^

We report a patient exposed to an unknown concentration of hydrogen chloride gas of a cleaning substance (HCl 22%) indoors in a room with no ventilation for an indefinite albeit short period. Despite being admitted in respiratory failure, her exposure occurred two days prior. She got progressively worse at home, complaining of dyspnea and persistent coughing. On arrival, the patient needed clinical support therapy focused on airway management, with supplemental oxygen and aerosolized bronchodilators, as needed.

β_2_ adrenergic agonist therapy is proposed as a means for relieving hydrochloric acid–induced bronchospasm. Some authorities recommend treatment with high doses of corticosteroids for patients with high-dose exposures, but this treatment’s value is questionable.^4^ Plausibly, corticosteroids could inhibit several of the involved pathophysiological processes such as (i) oxidative stress, (ii) increased lipid peroxidation, (iii) increased pro-inflammatory mediators, and/or (iv) changed cellular ultrastructures.^10^

In our case, the initial measures for bronchospasm were insufficient, and the patient required orotracheal intubation and mechanical ventilation. The patient had acute mild hypoxemia (PaO_2_/ fraction of inspired oxygen ratio between 200 and 300), bilateral opacities on chest radiography, not explained by cardiac failure or fluid overload. Thus, we considered acute respiratory distress syndrome diagnosis and adopted lung-protective ventilation strategies with low tidal volumes and limited plateau pressures.^15^ Some severely exposed patients may evolve with acute respiratory distress syndrome, and its full development takes time. Toxic reactive intermediates continue to be formed even after the exposure has ceased.^10^

The persistence of intermittent asthma symptoms after the accident, usually triggered by exposure to irritants, suggests Reactive Airway Dysfunction Syndrome (RADS). It is characterized by the appearance of asthma-like symptoms and an increase in nonspecific bronchial responsiveness after massive acute exposure to irritants. These individuals have a hyperreactive state, which may last from months to years, and persistent airway hyperresponsiveness is likely related, in this condition, to persistent epithelial damage, inflammation, and/or structural changes.^16^ ^,^ ^17^

The most probable pathogenesis of acute single exposure RADS and chronic irritant-induced asthma is bronchial or bronchiolar mucosa chemical irritation or burn. This is supported by biopsy studies in two initial case series by Brooks et al.^18^ How this mucosal inflammatory response leads to chronic and, in many cases, permanent airway dysfunction is not clear. The diagnosis of both acute and chronic irritant-induced asthma is based on history, supplemented by the measurement of the irritant’s level. It may be difficult to differentiate between chronic irritant-induced asthma and irritant-induced bronchoconstriction in patients with pre-existing asthma.^19^ ^,^ ^20^

The American College of Chest Physicians consensus criteria for the diagnosis of RADS was agreed in 1995,^21^ and are: (i) the absence of previous respiratory symptoms; (ii) the possibility for the patient to date the onset with a specific event; (iii) a high-level exposure to gas, (iv) smoke, (v) fume or vapor; (vi) the onset of symptoms occurring within a few hours after exposure with persistence for at least three months; (vii) asthma-like symptoms such as cough, wheezing, and dyspnea; (viii) pulmonary function tests that usually show airflow obstruction and increased response to methacholine; and (ix) no evidence of other pulmonary diseases.^16^

In this report, the patient was exposed to an environment containing an unknown concentration of HCl gas of a cleaning substance (HCl 22%). She had no personal or familial history of asthma or atopy, or respiratory symptoms prior to the HCl exposure, despite her smoker history. She also had no evidence of allergy or eosinophilia. The lung function test suggests mild obstructive ventilatory disorder responsive to bronchodilators. The sequence of events fits the pattern described in RADS. We had no histological biopsies. This patient fulfills all the necessary criteria for RADS.

RADS is a controversial and poorly understood condition. Traditional treatment is the same as conventional asthma, despite evidence that patients with RADS are less responsive to β_2_ agonists.^3^ ^,^ ^16^ ^,^ ^17^ ^,^ ^22^ The long-term outcome is not well documented, but if symptoms do not remit within six to twenty-four months, they are likely to remain persistent. In these cases, effective treatments are lacking, and the quality of life becomes impaired. Oral corticosteroids and bronchodilators started within the first three months of symptoms have had the most favorable outcomes.^3^

## CONCLUSION

The current guidelines on human exposures to Hydrogen Chloride (HCl) depend on specialist opinion rather than evidence-based medicine. The data used for establishing the dose and the related threshold effects are limited, and it is still difficult to extrapolate to non-occupational settings and the general public. Well-designed studies are necessary to understand better the HCl exposure-response information, its health effects, and evidence-based treatments to reduce patient morbidity.

High-level exposure to HCl may result in severe acute symptoms and long term chronic persistent effects. This leads to increased health system cost readmissions and patient morbidity. Most Reactive Airway Dysfunction Syndrome (RADS) cases are recognized retrospectively, and they usually lack accurate assessment of the exposure intensity. Because there are few cases described in the literature, effective treatment guidance lacks, especially for those with persistent symptoms after six months. RADS may be a predictor of lung disease progression in those chronic patients and an independent risk factor for irreversible loss of lung function.
